# Application of liquid biopsy for surgical management of pancreatic cancer

**DOI:** 10.1002/ags3.12317

**Published:** 2020-02-12

**Authors:** Minako Nagai, Masayuki Sho, Takahiro Akahori, Kenji Nakagawa, Kota Nakamura

**Affiliations:** ^1^ Department of Surgery Nara Medical University Kashihara Japan

**Keywords:** circulating tumor cells, circulating tumor DNA, exosomes, liquid biopsy, pancreatic ductal adenocarcinoma

## Abstract

Pancreatic ductal adenocarcinoma (PDAC) is one of the deadliest forms of cancer. Although drug development over the past decade has gradually improved the prognosis of PDAC, the prognosis remains extremely poor. The predominant determinant of a poor prognosis is that patients are already at the advanced stage when they are diagnosed. Therefore, it is essential to detect early‐stage PDAC to ensure a good prognosis. However, in general, being asymptomatic at the early stage makes the detection of early‐stage PDAC very difficult. Recently, much attention has been focused on the utility of a liquid biopsy as a biomarker. Theoretically, early‐stage tumors can be detected even under asymptomatic conditions. A number of studies on liquid biopsies have been reported so far. Several biomarkers, including circulating tumor DNA (ctDNA), circulating tumor cells (CTCS), and exosomes, are used in liquid biopsies, with the potential to be applied to the clinical setting. Each biomarker is reported to have different utilities, such as the detection of early‐stage disease, the differential diagnosis of PDAC from other types of pancreatic tumors, the prediction of the prognosis or risk of recurrence, and monitoring the treatment response. In this review, we focus on ctDNA, CTCS, and exosomes as representative liquid biopsy biomarkers and describe the specific functions of each biomarker in the treatment of PDAC. Furthermore, we discuss the application of liquid biopsies, especially for the surgical management of PDAC.

## Graphical abstract

Much attention has been focused on the utility of a liquid biopsy as a biomarker. We focus on ctDNA, CTCS, and exosomes as representative liquid biopsy biomarkers and describe the specific functions of each biomarker in the treatment of PDAC. Furthermore, we discuss the application of liquid biopsies, especially for the surgical management of PDAC.

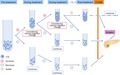

## 1 INTRODUCTION

Pancreatic cancer is one of the deadliest cancers. Of the 18 million cancer cases diagnosed around the world in 2018, nearly half a million were estimated to be pancreatic cancer.^1^ Despite many studies having been conducted globally to improve the prognosis of pancreatic cancer, the prognosis remains unsatisfactory. In general, more than half of patients with pancreatic cancer are diagnosed at the late stage because of early‐stage changes being asymptomatic. Therefore, the early detection is essential for improving the prognosis of pancreatic cancer.

Plasma protein markers, such as carbohydrate antigen 19‐9 (CA19‐9) and carcinoembryonic antigen (CEA), are commonly used. However, while they are reliable and noninvasive, they are insufficient to diagnose early‐stage pancreatic cancer, since these serum levels are also elevated in patients with inflammation and a smoking history, and their findings are sometimes normal even in the presence of metastasis.^2^

Recently, liquid biopsies have attracted attention for making an early diagnosis. Such biopsies can be collected from various sources, such as the blood, urine, pancreatic juice, and saliva. They also have many advantages over conventional tissue biopsies.^3^, ^4^ Conventional sampling of pancreatic tissue is sometimes harmful to the patients and can be challenging depending on the patient's general condition, tumor location, and bleeding tendency. In contrast, liquid biopsies are performed noninvasively.^5^ In addition, it is possible to repeatedly confirm the tumor properties using liquid biopsies in cases of tumor heterogeneity^6^, ^7^ and changes in response to treatment or surgery.^8^ A liquid biopsy is thus considered theoretically useful not only for making an early diagnosis but also for predicting the prognosis, performing longitudinal monitoring, and assessing the therapeutic effect.

The circulating cancer biomarkers include circulating tumor DNA (ctDNA), circulating tumor cells (CTCs), and exosomes, each of which has different potential as a cancer biomarker. In this review, we discuss how we can use a liquid biopsy in the surgical management of pancreatic ductal adenocarcinoma (PDAC).

## 2 CIRCULATING TUMOR DNA

**Figure 1:**
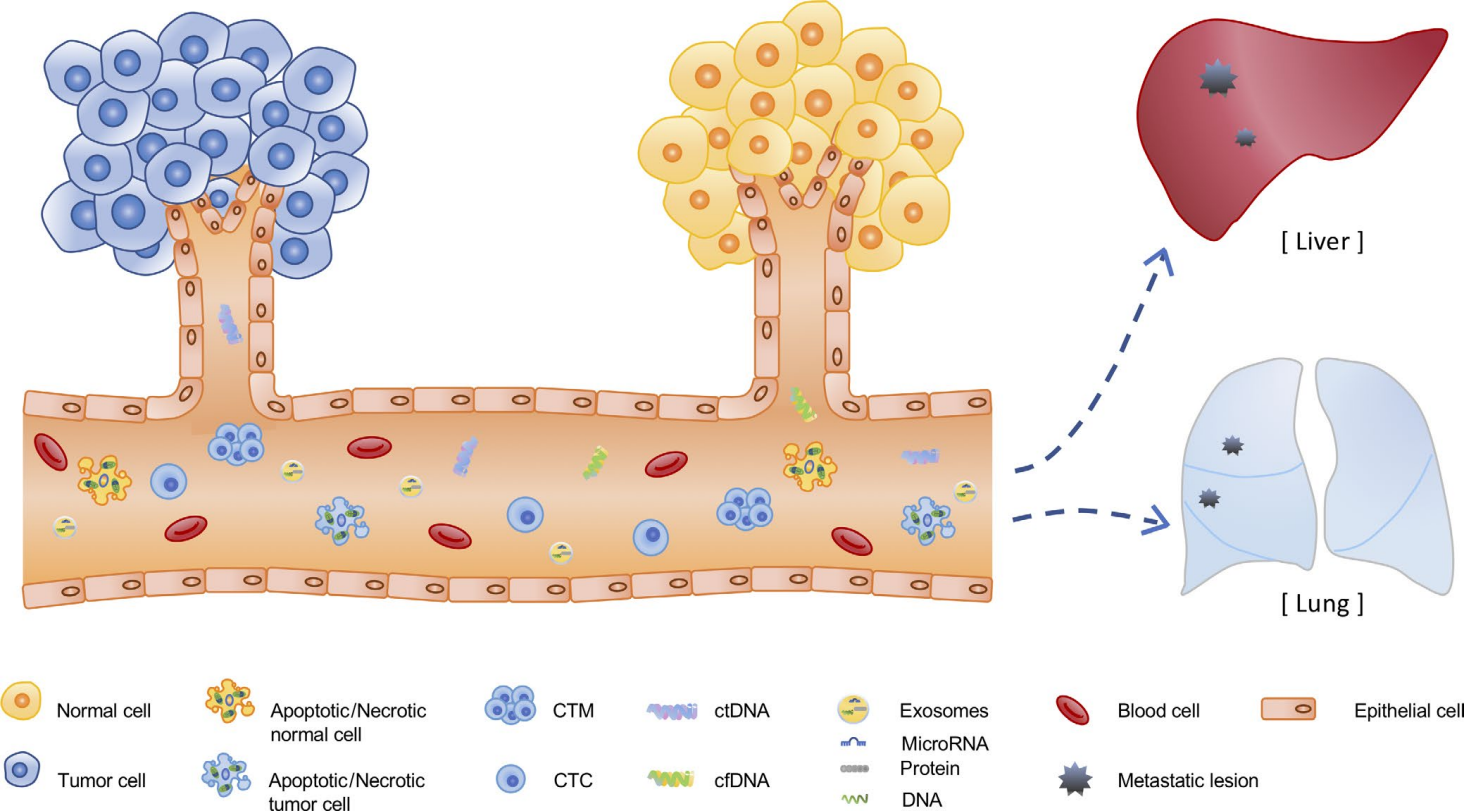
The cells shed various materials into the body fluid. The tumor‐derived components such as circulating tumor cells (CTCs), circulating tumor DNA (ctDNA), exosomes are released from tumor cells, as well as apoptotic or necrotic cells. And they are correlated with the formation of distant metastasis. They are useful as biomarkers for liquid biopsy because they contain the tumor genetic information

Circulating cell‐free DNA (cfDNA) is released into the plasma through various cellular physiological events, such as apoptosis, necrosis, and secretion (Figure 1).^9^, ^10^ In general, patients with cancer have much more normal circulating cfDNA than healthy individuals.^11^ Among them, cfDNA derived from tumor cells is called ctDNA.

The identification of oncogenic mutations with a high prevalence enables us to detect ctDNA from the pool of cfDNA. The detection of mutations in the Kirsten rat sarcoma (KRAS) gene in PDAC tissues obtained at an autopsy or surgically removed was reported in 1988.^12^ The KRAS mutation in blood from a pancreatic cancer patient was first detected using a liquid biopsy technique in 1994.^13^ The KRAS mutation occurs early during carcinogenesis^14^ and is observed in more than 90% of PDAC cases. Furthermore, it has been revealed that the mutations in three other genes—cyclin dependent kinase inhibitor 2A (CDKN2A), tumor protein 53 (TP53), and SMAD family member4 (SMAD4)/Deleted in Pancreatic Cancer‐4 (DPC4)—are frequently observed in PDAC tissues.^15^, ^16^, ^17^ These four gene mutations, which are often observed in PDAC, were relatively frequently detected than other genes also in ctDNA of PDAC patients.^18^ Most especially, KRAS mutation was reported to be observed in more than 80% of PDAC patients with detectable ctDNA.^19^, ^20^ On the other hand, potentially targetable somatic mutations were reported to be observed in approximately 30% of patients.^18^ Thus, the release of ctDNA from PDAC provides various genetic information. However, further studies are required in order to utilize such potentially valuable information for molecular‐targeted therapy for PDAC.

### 2.1 The development of analysis technology

To detect ctDNA from a liquid biopsy, it is essential to develop appropriate analysis techniques, such as allele‐specific polymerase chain reaction (PCR), digital PCR (dPCR), droplet digital PCR (ddPCR), and beads, emulsion, amplification, magnetics (BEAMing). These methods can achieve results with high sensitivity, quick speed, and relatively low cost. However, only known mutations can be detected.

Recently, next‐generation sequencing (NGS) technology has made the analysis of a large number of loci possible with high depth and sensitivity as well as the identification of unknown aberrations. This enabled us to cope with the heterogeneity issue of PDAC. However, a certain amount of DNA is required in order to perform a target sequence analysis using NGS, and only a small amount of ctDNA is contained in plasma. Therefore, the detection of ctDNA is generally difficult, even with NGS.^18^, ^20^, ^21^ To overcome the limitations of current technologies, a combination of different modalities, such as dPCR and NGS, has been used for the analysis of liquid biopsy and made it possible to analyze more efficiently.^18^ Further technology development may lead to breakthroughs in daily clinical practice.

### 2.2 Difficulty detecting ctDNA in early‐stage cancer

Circulating tumor DNA came to be considered a suitable cancer biomarker due to the identification of KRAS mutations in ctDNA from the blood of PDAC patients.^13^ The comprehensive genomic analysis for the cancers was expected to prove useful as a novel molecular diagnostic method. However, since ctDNA exists only in a small fraction (<1.0%) of total cfDNA,^6^ it had been challenging to detect tumor‐derived ctDNA. Takai et al^18^ reported that KRAS mutations were detected more sensitively in PDAC patients with metastasis than in those without metastasis. Quantitative information of ctDNA reflects the tumor burden,^22^ indicating the difficulty of making a diagnosis of early‐stage cancer with a low tumor burden.

Once cfDNA is released into the circulation, it is immediately cleared from the circulation by nuclease action and urinary excretion. Furthermore, its uptake by the liver and spleen and degradation by macrophages may also influence elimination from circulation.^23^, ^24^ Therefore the half‐life of ctDNA in the circulation is very short, at most a few hours.^23^ This short half‐life makes it challenging to detect ctDNA in cases of early‐stage cancer.

The blood is considered the most common body fluid source for liquid biopsies to detect PDAC. However, the pancreatic juice is also an effective source for detecting PDAC.^25^, ^26^ Yu et al^25^ performed a genetic analysis to detect pancreatic ductal neoplasia using pancreatic juice. The mutant DNA concentrations were significantly higher in PDAC than in intraductal papillary mucinous neoplasm (IPMN). Furthermore, TP53 and/or SMAD4 mutations were commonly detected in PDAC and were not detected in controls. Although the specificity was high at 100%, the sensitivity was low at 32%. Interestingly, a few PDAC patients had SMAD4/TP53 mutations in the pancreatic juice collected more than 1 year before they were diagnosed with PDAC.^25^ Furthermore, Kisiel et al^26^ reported that methylation signature in pancreatic juice enables PDAC to be distinguished from normal or chronic pancreatitis. Although the procedure for collecting pancreatic juice is complicated and harmful to patients, multiple liquid biopsy biomarkers from the pancreatic juice may help us diagnose early‐stage pancreatic cancer.

### 2.3 Promising biomarkers for predicting the prognosis

It is difficult to detect ctDNA in early‐stage pancreatic cancer because of the low abundance of ctDNA in the early stage. In contrast, the ctDNA can be detected with high probability in the plasma of advanced pancreatic cancer patients with metastasis.^18^, ^20^ A high abundance of ctDNA is significantly associated with a poor prognosis.^18^, ^19^, ^20^

Hadano et al^19^ assessed the prognosis of patients who underwent curative pancreatoduodenectomy for PDAC according to the presence of KRAS‐mutated ctDNA in the plasma. The median overall survival of the patients with ctDNA was significantly worse than that of those without ctDNA (13.6 and 27.6 months respectively). They further showed that the presence of ctDNA in the preoperative plasma was associated with a poor prognosis. In addition, Pietrasz et al^20^ reported that patients with ctDNA detectable by at least one cancer‐specific gene mutation, such as KRAS, TP53, and SMAD4, in the plasma collected after curative surgery had a significantly worse prognosis and shorter disease‐free survival than those with undetectable ctDNA. Progression was detected earlier using ctDNA than computed tomography (CT). Therefore, ctDNA may be a promising pancreatic cancer biomarker for predicting the postoperative prognosis.

If ctDNA is detected in the plasma collected after surgery, it may be better to consider a stronger regimen of adjuvant therapy. This effective stratification might help provide sufficient adjuvant treatment for high‐risk patients and avoid excess treatment for low‐risk patients.

### 2.4 Biomarker for predicting the treatment response and longitudinal monitoring

The detection of ctDNA is helpful for real‐time monitoring of PDAC during treatment. Melani et al^27^ reported that the changes in the ctDNA collected from patients with lymphoma were correlated with positive responses to chemotherapy for lymphoma. In addition, recurrence was able to be detected using ctDNA months before it was noted by CT. ctDNA is therefore useful for not only assessing the treatment response during treatment but also longitudinal monitoring during surveillance.^27^ Furthermore, if ctDNA is detected in preoperative blood samples from patients with pancreatic cancer, unnecessary laparotomy may be avoided, and it may help us choose chemotherapy before surgery.

## 3 CIRCULATING TUMOR CELLS

In general, cancer originates from the epithelial tissue located on the outermost surface of the tissue and invades the surrounding tissues through the basement membrane. The cancer cells then penetrate into the lymphatic and blood vessels from the primary tumor, circulate to the whole body, and finally connect with the surrounding tissue and other organs, resulting in metastasis. The cancer cells that invade the blood vessels during the process of metastasis are CTCs and exist singly or in the form of cell clusters, namely circulating tumor microemboli (CTM) (Figure 1). CTM have been reported to have a higher survival and migration ability than CTCs.^28^

Cancer cells initially retain the characteristics of the epithelial cells from which they originate. They then develop various phenotypes to avoid apoptosis and form metastatic lesions. Epithelial‐to‐mesenchymal transition (EMT) is a key process in cancer initiation and progression.^29^, ^30^ Through EMT, CTCs improve their migration and invasion ability. The molecules expressed on CTCs, including epithelial cell adhesion molecule (EpCAM) and E‐cadherin, may also change during EMT.

### 3.1 Methods of detecting CTCs

Circulating tumor cells account for only a few of the billions of blood cells present in 1 mL of blood. Therefore, enrichment and identification are needed for the detection of CTCs. The isolation strategies of CTCs exploit biological properties (expression and cell surface markers) and physical properties (density and size). The immunoaffinity capture methods use epithelial markers and magnetic beads conjugated to antibodies recognizing CTCs. This technique is based on the capture of epithelial markers, such as EpCAM. This enrichment method is the most commonly used; however, since many CTCs do not express EpCAM because of EMT, the counts might be underestimated. CTCs without EpCAM expression can be enriched indirectly by capturing white blood cells instead of CTCs. CTCs are also enriched by a method that exploits the activation of telomerase.^31^

Other methods using physical properties include density gradient centrifugation and size‐exclusion chromatography. Furthermore, combined methods involving both biological and physical properties are sometimes used. To date, many approaches have been developed to increase the detection rate of CTCs.

The detection rate reportedly varies depending on the blood vessels from which the blood is collected. Catenacci et al^32^ reported that CTCs in the portal blood could be detected with a 100% sensitivity in patients with pancreaticobiliary cancers, while those in the peripheral blood were detected with only a 22% sensitivity. In addition, Tien et al^33^ reported that CTCs in portal blood were predictive of liver metastasis. These studies suggest the importance of the source of blood samples.

### 3.2 Potential predictive biomarker for early‐stage pancreatic cancer

Many studies have shown that CTCs could be detected in early‐stage pancreatic cancer patients.^8^, ^32^, ^34^, ^35^ The sensitivity was relatively high at more than 70%.^36^ In addition, CTCs were detected even in pre‐malignancy cases.^8^, ^32^ However, some studies have reported that the detection rate of CTCs was not high in cases of early‐stage pancreatic cancer, with a sensitivity of <50%.^37^, ^38^ It is therefore necessary to establish a more reliable method for detecting early‐stage pancreatic cancer, since the sensitivity of current methods is not high enough for practical applications.

### 3.3 Promising biomarkers for predicting the prognosis and recurrence

Circulating tumor cells are hardly detected in healthy people.^39^, ^40^, ^41^ The presence of CTCs usually indicates advanced‐stage disease and is correlated with a poor prognosis in various types of cancers.^35^, ^39^, ^40^, ^42^ Han et al^42^ reported that PDAC patients with CTCs had a worse progression‐free and overall survival than those without CTCs in a meta‐analysis of nine cohort studies. In addition, a subgroup analysis by ethnicity indicated that patients with CTCs had a poor prognosis among Asian and Caucasian populations.^42^ Those studies suggested that CTCs might be a promising prognostic marker. Patients with metastatic prostate and breast cancer showed CTCs co‐expressing epithelial and mesenchymal markers because of EMT.^43^ The presence of CTCs co‐expressing both vimentin and cytokeratin was reported to be associated with recurrence.^34^, ^41^, ^42^ Therefore, further studies on CTCs in PDAC are clearly warranted.

### 3.4 Indicators of chemotherapeutic efficacy and monitoring

Circulating tumor cell counts are known to be increased in late‐stage disease. Indeed, Ankeny et al^37^ reported that patients who had occult metastasis of pancreatic cancer had significantly more CTCs than those without metastasis. These findings may suggest that we should consider systemic chemotherapy instead of upfront surgery if we detect elevated CTC counts in the blood of PDAC patients even though the metastasis is not detected on imaging.

**Figure 2:**
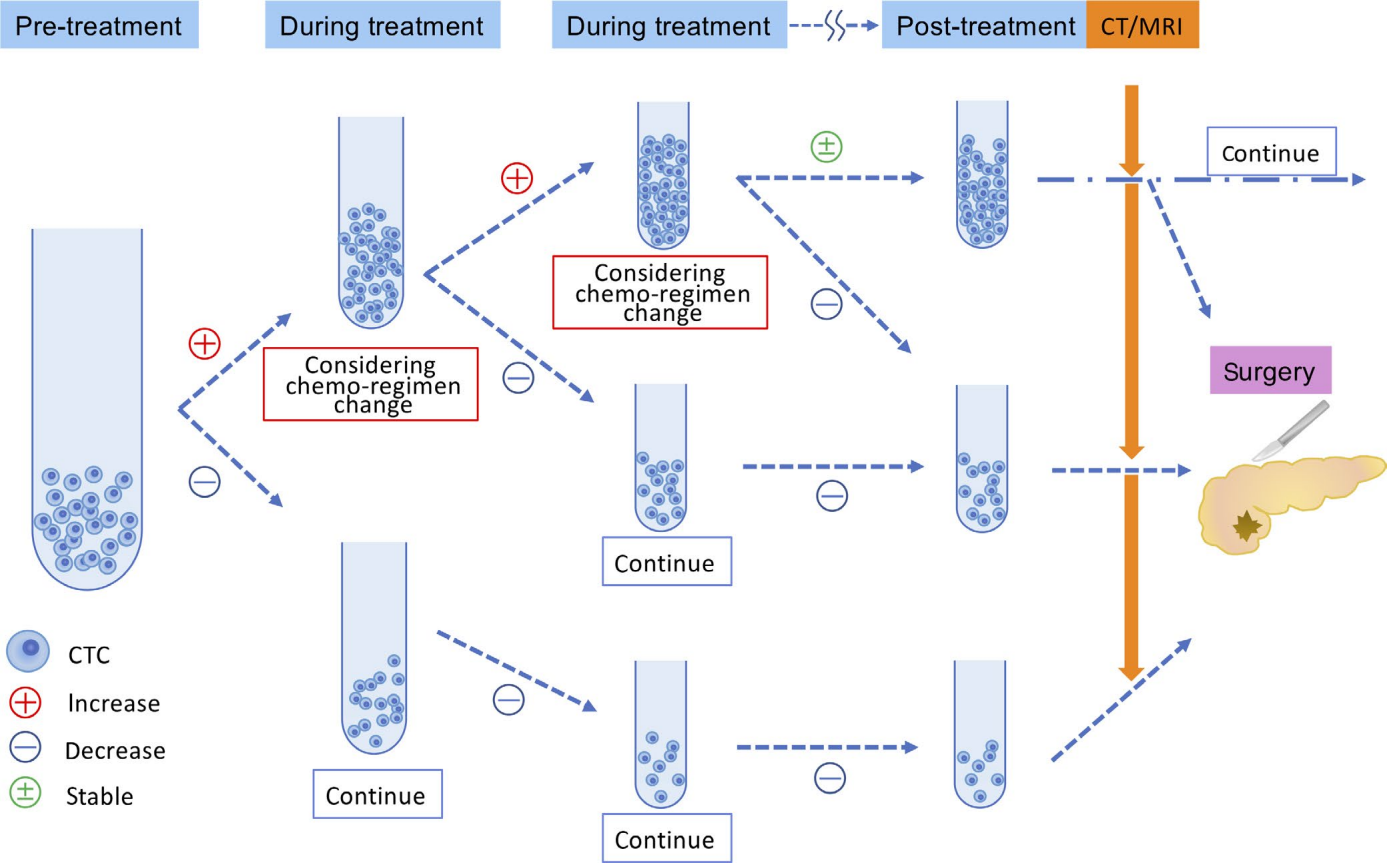
CTCs reflect therapeutic response sensitively. If we evaluate the therapeutic effect frequently during treatment by examining CTCs as a biomarker, it may be possible to change the chemotherapy regimen without delay, and the patients will not miss the opportunity to undergo surgery

Recently, the efficacy of neoadjuvant treatment (NAT) for not only borderline pancreatic cancer but also resectable pancreatic cancer has been reported.^44^, ^45^, ^46^ The therapeutic effect of NAT is usually confirmed by CT and protein biomarkers after the end of the scheduled treatment. However, some PDAC patients receive no therapeutic benefit from NAT, and their tumors progress during therapy. Such patients may thus risk missing the chance to undergo curative surgery. CTC measurements may help avoid such unfavorable clinical outcomes. Furthermore, CTM have been reported to be suitable for assessing the chemo‐response in patients with advanced pancreatic cancer.^8^ Therefore, the therapeutic effect may be able to be evaluated more sensitively by examining CTCs or CTM frequently during preoperative treatment. For instance, if CTC counts are increased, it may be better to consider changing the chemotherapy regimen immediately (Figure 2).

If a CT image shows solid soft tissue or increased hazy attenuation around an artery without distant metastasis, the case may be diagnosed as locally advanced pancreatic cancer with artery invasion. Even if anti‐cancer treatment is very effective pathologically, these changes on CT images sometimes remain. If CTCs are used to monitor the therapeutic effect, a more appropriate treatment can be provided at a suitable timing. This means that patients will not miss the opportunity to undergo surgery and that may improve their prognosis. Even for patients with metastasis cancer, it may be possible to change the chemotherapy regimen without delay based on a CTC examination instead of a regular CT examination. Taken together, these findings suggest that CTCs might be a promising monitoring marker.

## 4 EXOSOMES

Exosomes are a type of extracellular vesicle with a diameter of 40‐150 nm that is secreted from all types of cells. They contain DNA, microRNA, and proteins on the inside and display proteins also on their surfaces (Figure 1). Exosomes secreted from cells circulate in the body fluids and communicate between cells. Those derived from tumors are expected to be useful for the diagnosis and as a biomarker.

Exosomes are isolated using their biological and physical properties, as mentioned above for CTCs. However, using biological methods, potentially important exosomes not expressing the selected markers may be overlooked. Furthermore, polymer precipitation methods may be limited by a high recovery with low purity.^47^ It is thus necessary to solve such problems for the clinical application and to standardize the detection methods.

### 4.1 Potential utility as predictive biomarkers for early‐stage pancreatic cancer

In 2014, tumor‐derived exosomes were introduced as novel biomarkers containing tumor‐specific DNA from PDAC.^48^ microRNA have demonstrated very high accuracy for discriminating PDAC patients from pancreatitis patients and healthy controls.^2^, ^49^ Recently, it was reported that microRNA is useful for detecting early‐stage cancer.^2^, ^49^, ^50^ Melo et al^50^ identified the cell surface proteoglycan glypican‐1 (GPC1) on tumor‐derived exosomes. GPC1 has also been reported to be useful for distinguishing healthy persons from patients with benign disease and patients with early‐ and late‐stage pancreatic cancer with high sensitivity and specificity.^2^, ^49^, ^50^ In addition, Lai et al^2^ showed that microRNA were useful for distinguishing PDAC from pancreatitis.

Using microRNA will help us make more accurate diagnoses, even if it is difficult to diagnose whether the pancreatic lesion is a tumor or pancreatitis on images or with other protein markers.

Furthermore, Vila‐Navarro et al^51^ showed that microRNA in cyst fluid was able to distinguish pancreatic cancer patients from healthy controls. Furthermore, Wang et al^52^ reported that miR‐216a and miR‐217 in cyst fluid was able to distinguish high‐grade dysplasia and PDAC from low‐grade dysplasia. The combination of pancreatic juice and imaging findings may lead to a more accurate diagnosis and the earlier performance of surgery.

### 4.2 Biomarkers for predicting the prognosis and monitoring after surgery

Melo et al^50^ also reported that the levels of GPC1 on tumor‐derived exosomes were associated with the tumor burden and survival of pre‐ and post‐surgical patients. Lai et al^2^ showed that elevated microRNA levels decreased to a normal level within 24 hours of pancreatic resection for PDAC and indicated the utility of microRNA for monitoring. The level of tumor‐derived exosomes is correlated with the stage and presence of metastasis.^50^, ^53^ Tumor‐derived exosomes can reflect the response to surgery and treatment^50^, ^54^ and are therefore considered to be promising prognostic and monitoring biomarkers of pancreatic cancer. Persistently high microRNA levels after surgery may suggest the presence of occult metastasis. In such cases, patients might need to be followed carefully and considered for more effective or long‐term adjuvant treatment after surgery.

### 4.3 The identification of metastatic location

Interestingly, it was reported that tumor‐derived exosomes with organ specificity were preferentially taken up by cells and prepared the metastatic niche. The expression of integrins on exosomes exhibited a pattern reflecting organ specificity. For example, Hoshino et al^55^ showed that α6β4 and α6β1 integrins on tumor‐derived exosomes were associated with lung metastasis, while αvβ5 integrins were associated with liver metastasis (Figure 1). Exosomal integrins in blood may therefore be useful for predicting organ‐specific metastasis.

Furthermore, by focusing on the function of exosomes as an intercellular communication tool their utility for drug delivery to therapeutic target cells is also gathering attention.^56^ Indeed, Kamerkar et al^57^ showed that engineered exosomes suppressed cancer in multiple PDAC mouse models and significantly improved the overall survival. The utility of exosomes in therapeutic applications will be explored in the future.

## 5 SUMMARY AND FUTURE PERSPECTIVES

In many reports on liquid biopsies, the sensitivity and specificity of the suggested biomarkers are insufficient with marked variation; both the false‐positive and false‐negative rates should be sufficiently low for the treatment of pancreatic cancer. Furthermore, the detection methods for biomarkers must be standardized.

However, if reliable liquid biopsies are successfully introduced in the future, the detection of early‐stage pancreatic cancer before the appearance of symptoms might be possible. This will strengthen the possibility of a cure for pancreatic cancer. In addition, if the precursor lesion can be detected by liquid biopsies, minimally invasive surgery, such as laparoscopic and robotic surgery, will surely become more widespread. A liquid biopsy is also potentially very useful for evaluating the therapeutic efficacy for advanced cancer, since it can theoretically be more sensitive than a conventional imaging study. If we could assess the therapeutic efficacy frequently using a liquid biopsy, we would be able to select the therapeutic strategy in a real‐time manner for each patient. Importantly, a liquid biopsy can be expected as a more cost‐effective method than conventional modality by avoiding ineffective medical treatment. On the other hand, current liquid biopsy takes time and is costly. Therefore, technological advancement is required.

## 6 CONCLUSIONS

Liquid biopsies hold great promise in the field of precision medicine and personalized treatments for pancreatic cancer patients. Although further studies on such biopsies are clearly needed, the development of novel technologies and targeted therapy may dramatically improve patient care and lead to a cure for this fatal disease.

## DISCLOSURE

Funding: None.

Conflict of Interest: The authors declare no conflicts of interest for this article.

Author Contribution: Masayuki Sho devised the main conceptual idea and proof outline. Minako Nagai selected and reviewed references, wrote the initial draft of this manuscript. Takahiro Akahori, Kenji Nakagawa, and Kota Nakamura helped review the references and assisted in the presentation of this manuscript.
